# Supplementary material for: Neonatal Hypoglycemia and Neurodevelopmental Outcomes—An Updated Systematic Review and Meta-Analysis
Source: Life (Basel). 2024 Dec 6;14(12):1618. doi: 10.3390/life14121618 (PMC11677687; doi:10.3390/life14121618)
Supplement: Supplementary file 1 [file life-14-01618-s001.zip › life-3289338-supplementary.pdf]

# Online supplementary material

## Table of Contents

1. Search Strategy
2. Forest Plots
3. Characteristics of excluded studies:
4. NOS tool [21]
5. GRADE Table [22]

**1 . Search strategy:** 7,687 results

"infant, newborn"[MeSH Terms] AND "hypoglycemia"[MeSH Terms]) OR "outcome assessment, health care"[MeSH Terms])

5,382 results
Search: **(newborn) AND (hypoglycemia)** Filters: **Newborn: birth-1 month**
(("infant, newborn"[MeSH Terms] OR ("infant"[All Fields] AND "newborn"[All Fields]) OR "newborn infant"[All Fields] OR "newborn"[All Fields] OR "newborns"[All Fields] OR "newborn s"[All Fields]) AND ("hypoglycaemia"[All Fields] OR "hypoglycemia"[MeSH Terms] OR "hypoglycemia"[All Fields] OR "hypoglycaemias"[All Fields] OR "hypoglycemias"[All Fields])) AND (newborn[Filter])

1,743 results
(("hypoglycaemia"[All Fields] OR "hypoglycemia"[MeSH Terms] OR "hypoglycemia"[All Fields] OR "hypoglycaemias"[All Fields] OR "hypoglycemias"[All Fields]) AND ("infant"[MeSH Terms] OR "infant"[All Fields] OR "infants"[All Fields] OR "infant s"[All Fields]) AND ("outcome"[All Fields] OR "outcomes"[All Fields])) AND (newborn[Filter])
**Translations**
**hypoglycemia:** "hypoglycaemia"[All Fields] OR "hypoglycemia"[MeSH Terms] OR "hypoglycemia"[All Fields] OR "hypoglycaemias"[All Fields] OR "hypoglycemias"[All Fields]
**infant:** "infant"[MeSH Terms] OR "infant"[All Fields] OR "infants"[All Fields] OR "infant's"[All Fields]
**outcome:** "outcome"[All Fields] OR "outcomes"[All Fields]

34 results

Search: **((neonatal[MeSH Terms]) AND (hypoglycemia[MeSH Terms])) AND (brain injuries[MeSH Terms])** Filters: **Newborn: birth-1 month**

("infant, newborn"[MeSH Terms] AND "hypoglycemia"[MeSH Terms] AND "brain injuries"[MeSH Terms]) AND (newborn[Filter])

**Translations**

**neonatal[MeSH Terms]:** "infant, newborn"[MeSH Terms]

**hypoglycemia[MeSH Terms]:** "hypoglycemia"[MeSH Terms]

**brain injuries[MeSH Terms]:** "brain injuries"[MeSH Terms]

## 2. Figure S1 A-G

### Secondary outcomes

1A. Hypoglycemia and Mild Cognitive Impairment

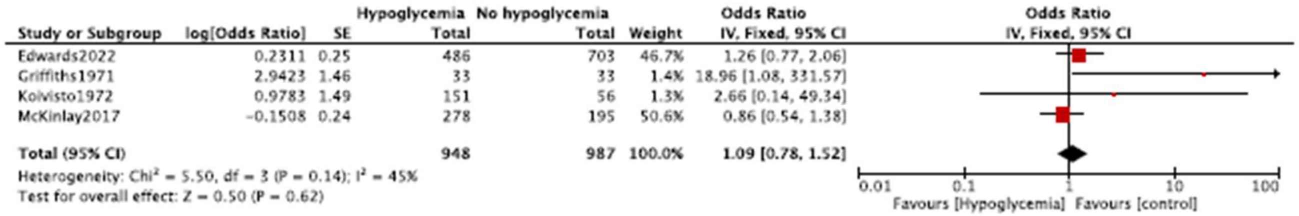


1B. Hypoglycemia and Moderate-Severe Cognitive Impairment

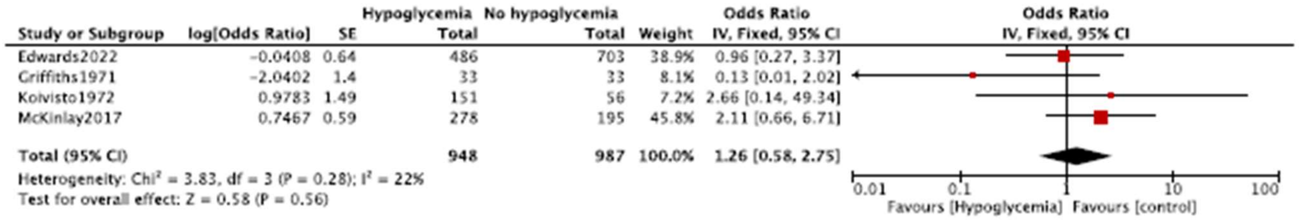

1C. Hypoglycemia and low-language or literacy

| Study or Subgroup | log[Odds Ratio] | SE | Hypoglycemia Total | No hypoglycemia Total | Weight | Odds Ratio IV, Fixed, 95% CI |
|---|---|---|---|---|---|---|
| Haworth1976 | 1.6544 | 1.51 | 25 | 12 | 100.0% | 5.23 [0.27, 100.88] |
| **Total (95% CI)** | | | **25** | **12** | **100.0%** | **5.23 [0.27, 100.88]** |

Heterogeneity: Not applicable
Test for overall effect: Z = 1.10 (P = 0.27)

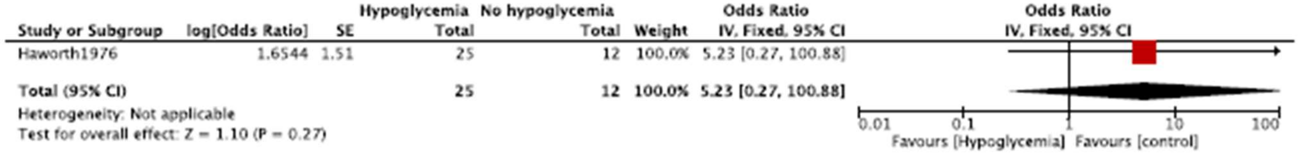


1D. Hypoglycemia and epilepsy

| Study or Subgroup | log[Odds Ratio] | SE | Hypoglycemia Total | No hypoglycemia Total | Weight | Odds Ratio IV, Fixed, 95% CI |
|---|---|---|---|---|---|---|
| Griffiths1971 | 0 | 0.84 | 41 | 41 | 30.5% | 1.00 [0.19, 5.19] |
| Haworth1976 | 0.9783 | 1.57 | 25 | 12 | 8.7% | 2.66 [0.12, 57.71] |
| Koivisto1972 | 0.9783 | 1.49 | 151 | 56 | 9.7% | 2.66 [0.14, 49.34] |
| McKinlay2017 | 0.9361 | 0.65 | 262 | 184 | 51.0% | 2.55 [0.71, 9.12] |
| **Total (95% CI)** | | | **479** | **293** | **100.0%** | **1.93 [0.78, 4.80]** |

Heterogeneity: $Chi^2 = 0.88$, df = 3 (P = 0.83); $I^2 = 0\%$
Test for overall effect: Z = 1.42 (P = 0.16)

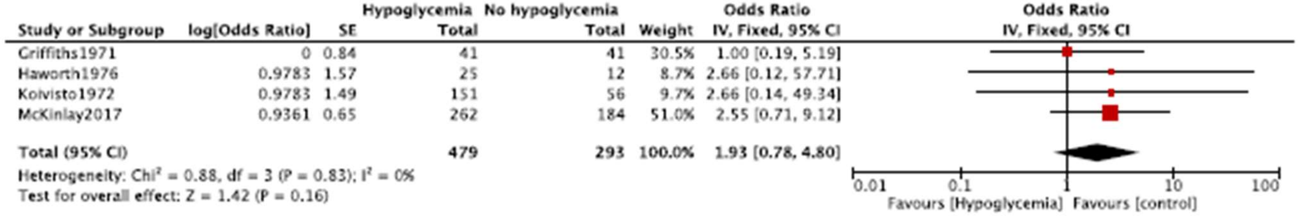


1E. Hypoglycemia and hearing impairment

| Study or Subgroup | log[Odds Ratio] | SE | Hypoglycemia Total | No hypoglycemia Total | Weight | Odds Ratio IV, Fixed, 95% CI |
|---|---|---|---|---|---|---|
| McKinlay2017 | −0.1625 | 0.18 | 268 | 188 | 100.0% | 0.85 [0.60, 1.21] |
| **Total (95% CI)** | | | **268** | **188** | **100.0%** | **0.85 [0.60, 1.21]** |

Heterogeneity: Not applicable
Test for overall effect: Z = 0.90 (P = 0.37)

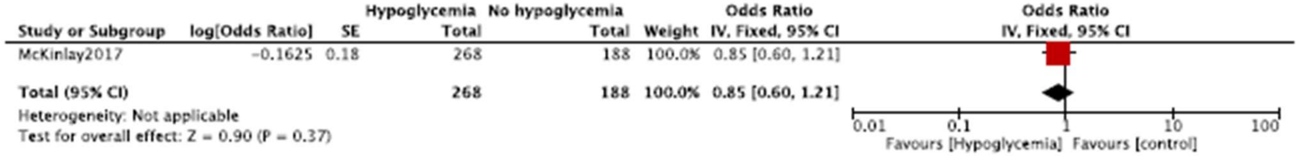

1F. Hypoglycemia and motor impairment

| Study or Subgroup | log[Odds Ratio] | SE | Hypoglycemia Total | No hypoglycemia Total | Weight | Odds Ratio IV, Fixed, 95% CI |
|---|---|---|---|---|---|---|
| Edwards2022 | 0.27 | 0.46 | 483 | 702 | 21.2% | 1.31 [0.53, 3.23] |
| Haworth1976 | -0.0408 | 1.26 | 25 | 12 | 2.8% | 0.96 [0.08, 11.34] |
| McKinlay2017 | 0.6043 | 0.39 | 272 | 187 | 29.4% | 1.83 [0.85, 3.93] |
| Wickstrom2018 | 0.3436 | 0.31 | 77 | 3294 | 46.6% | 1.41 [0.77, 2.59] |
| **Total (95% CI)** | | | **857** | **4195** | **100.0%** | **1.48 [0.98, 2.24]** |

Heterogeneity: $Chi^2 = 0.51, df = 3 (P = 0.92); I^2 = 0\%$
Test for overall effect: $Z = 1.86 (P = 0.06)$

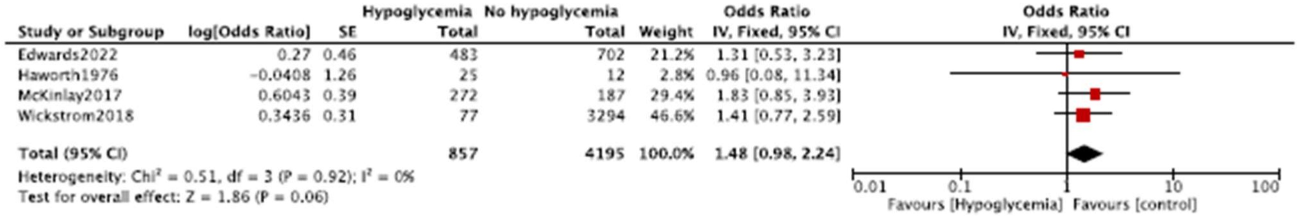


1G. Hypoglycemia and emotional-behavioural difficulties

| Study or Subgroup | log[Odds Ratio] | SE | Hypoglycemia Total | No hypoglycemia Total | Weight | Odds Ratio IV, Fixed, 95% CI |
|---|---|---|---|---|---|---|
| Griffiths1971 | -0.4005 | 0.62 | 41 | 41 | 6.9% | 0.67 [0.20, 2.26] |
| Haworth1976 | 0.9783 | 1.57 | 25 | 12 | 1.1% | 2.66 [0.12, 57.71] |
| McKinlay2017 | 0.0296 | 0.17 | 273 | 195 | 92.0% | 1.03 [0.74, 1.44] |
| **Total (95% CI)** | | | **339** | **248** | **100.0%** | **1.01 [0.73, 1.39]** |

Heterogeneity: $Chi^2 = 0.83, df = 2 (P = 0.66); I^2 = 0\%$
Test for overall effect: $Z = 0.06 (P = 0.95)$

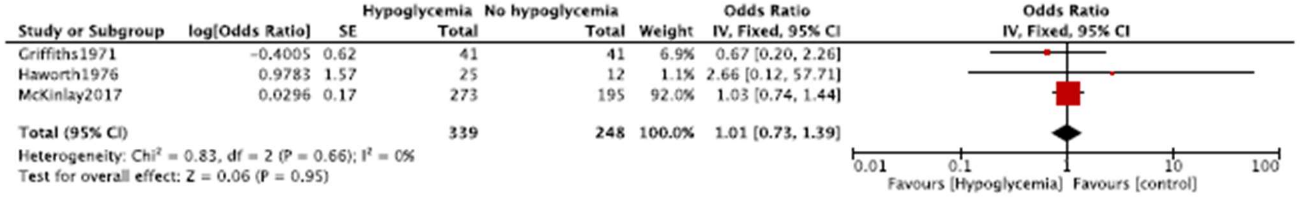


**Figure S2 A-C: Sensitivity analyses based on different blood glucose cut-offs**

2A. Severe hypoglycemia (BG <20mg/dl) and NDI (2-5 years)

| Study or Subgroup | log[Odds Ratio] | SE | Hypoglycemia Total | Control Total | Weight | Odds Ratio IV, Fixed, 95% CI |
|---|---|---|---|---|---|---|
| Haworth1976 | -0.2485 | 0.73 | 37 | 37 | 47.2% | 0.78 [0.19, 3.26] |
| Pildes1974 | 0.1044 | 0.69 | 17 | 16 | 52.8% | 1.11 [0.29, 4.29] |
| **Total (95% CI)** | | | **54** | **53** | **100.0%** | **0.94 [0.35, 2.51]** |

Heterogeneity: $Chi^2 = 0.12, df = 1 (P = 0.73); I^2 = 0\%$
Test for overall effect: $Z = 0.12 (P = 0.90)$

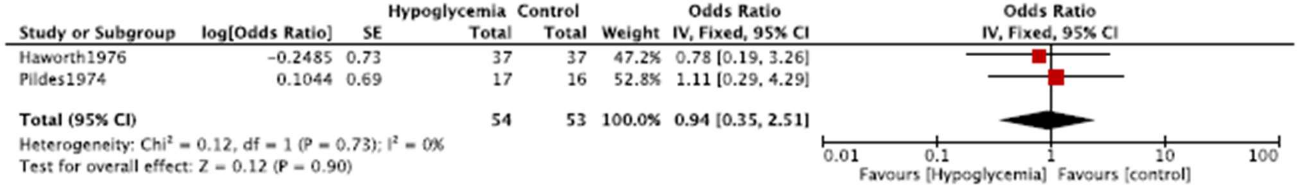

2B. Severe hypoglycemia (BG <20mg/dl) and epilepsy

| Study or Subgroup | log[Odds Ratio] | SE | Hypoglycemia Total | Control Total | Weight | Odds Ratio IV, Fixed, 95% CI |
|---|---|---|---|---|---|---|
| Griffiths1971 | 0 | 0.83 | 41 | 41 | 77.9% | 1.00 [0.20, 5.09] |
| Haworth1976 | 0.9783 | 1.56 | 25 | 12 | 22.1% | 2.66 [0.13, 56.59] |
| **Total (95% CI)** | | | **66** | **53** | **100.0%** | **1.24 [0.30, 5.22]** |

Heterogeneity: $Chi^2 = 0.31$, $df = 1$ ($P = 0.58$); $I^2 = 0\%$
Test for overall effect: $Z = 0.29$ ($P = 0.77$)

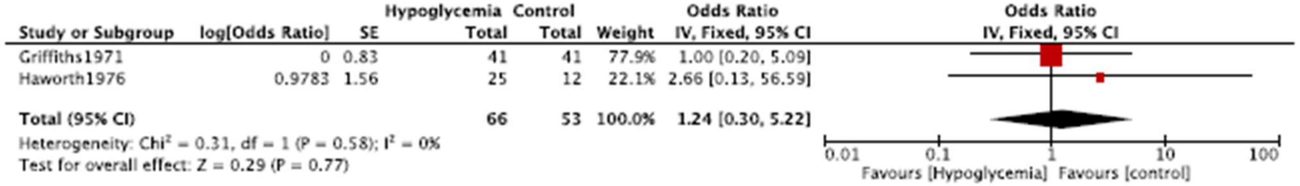


2C. Severe hypoglycemia (BG<20mg/dl) and emotional - behavioural difficulties

| Study or Subgroup | log[Odds Ratio] | SE | Hypoglycemia Total | Control Total | Weight | Odds Ratio IV, Fixed, 95% CI |
|---|---|---|---|---|---|---|
| Griffiths1971 | -0.4005 | 0.62 | 41 | 41 | 86.4% | 0.67 [0.20, 2.26] |
| Haworth1976 | 0.9783 | 1.56 | 25 | 12 | 13.6% | 2.66 [0.13, 56.59] |
| **Total (95% CI)** | | | **66** | **53** | **100.0%** | **0.81 [0.26, 2.50]** |

Heterogeneity: $Chi^2 = 0.67$, $df = 1$ ($P = 0.41$); $I^2 = 0\%$
Test for overall effect: $Z = 0.37$ ($P = 0.71$)

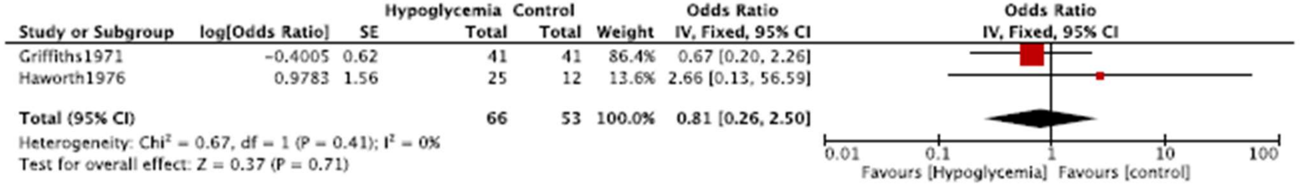

## 3. List of excluded studies:


1. Arhan, E., Öztürk, Z., Serdaroğlu, A., Aydın, K., Hirfanoğlu, T., & Akbaş, Y. (2017). Neonatal hypoglycemia: A wide range of electroclinical manifestations and seizure outcomes. *European journal of paediatric neurology : EJPN : official journal of the European Paediatric Neurology Society*, *21*(5), 738–744. https://doi.org/10.1016/j.ejpn.2017.05.009

2. Tottman, A. C., Alsweiler, J. M., Bloomfield, F. H., Pan, M., & Harding, J. E. (2017). Relationship between Measures of Neonatal Glycemia, Neonatal Illness, and 2-Year Outcomes in Very Preterm Infants. *The Journal of pediatrics*, *188*, 115–121. https://doi.org/10.1016/j.jpeds.2017.05.052

3. Vain, N. E., & Chiarelli, F. (2021). Neonatal Hypoglycaemia: A Never-Ending Story?. *Neonatology*, *118*(5), 522–529. https://doi.org/10.1159/000514711

4. Uyur Yalçın E, Maraş Genç H, Bayhan A, Anık Y, Kara B. Neurodevelopmental Outcome in Patients with Typical Imaging Features of Injury as a Result of Neonatal Hypoglycemia. Arch Neuropsychiatry 2022;59:296−302.

5. Tam, E. W., Haeusslein, L. A., Bonifacio, S. L., Glass, H. C., Rogers, E. E., Jeremy, R. J., Barkovich, A. J., & Ferriero, D. M. (2012). Hypoglycemia is associated with increased risk for brain injury and adverse neurodevelopmental outcome in neonates at risk for encephalopathy. *The Journal of pediatrics*, *161*(1), 88–93. https://doi.org/10.1016/j.jpeds.2011.12.047

6. Paulsen, M. E., & Rao, R. B. (2022). Cerebral Effects of Neonatal Dysglycemia. *Clinics in perinatology*, *49*(2), 405–426. https://doi.org/10.1016/j.clp.2022.02.008

7. Giouleka S, Gkiouleka M, Tsakiridis I, Daniilidou A, Mamopoulos A, Athanasiadis A, Dagklis T. Diagnosis and Management of Neonatal Hypoglycemia: A Comprehensive Review of Guidelines. Children (Basel). 2023 Jul 14;10(7):1220. doi: 10.3390/children10071220. PMID: 37508719; PMCID: PMC10378472.

8. Kryštof Tabery, Miloš Černý, Krzysztof Urbaniec, Miroslav Vaniš, Petr Zoban & Kateřina Štechová (2020) Continuous glucose monitoring as a screening tool for neonatal hypoglycemia in infants of diabetic mothers, The Journal of Maternal-Fetal & Neonatal Medicine, 33:11, 1889-1894, DOI: 10.1080/14767058.2018.1533941

9. Hoermann H, Roeper M, Dafsari RS, et al. Protecting against brain damage by improving treatment in neonates with hypoglycaemia: ProBrain- D—a study protocol of a prospective longitudinal study. BMJ Open 2022;12:e063009. doi:10.1136/ bmjopen-2022-063009

10. Kryštof Tabery, Ladislava Doležalová, Miloš Černý, Jan Janota, Petr Zoban & Kateřina Štechová (2022) Feasibility and Safety of Continuous Glucose Monitoring in Infants at Risk of Hypoglycemia in a Rooming-in Setting, Fetal and Pediatric Pathology, 41:4, 627-633, DOI: 10.1080/15513815.2021.1945716

11. Fluge G. (1975). Neurological findings at follow-up in neonatal hypoglycaemia. *Acta paediatrica Scandinavica*, *64*(4), 629–634. https://doi.org/10.1111/j.1651-2227.1975.tb03894.x

12. Galderisi A, Trevisanuto D, Russo C, Hall R, Bruschettini M. Continuous glucose monitoring for the prevention of morbidity and mortality in preterm infants. Cochrane Database Syst Rev. 2021 Dec 21;12(12):CD013309. doi: 10.1002/14651858.CD013309.pub3. PMID: 34931697; PMCID: PMC8690212.

13. Griffith, R., Hegarty, J. E., Alsweiler, J. M., Gamble, G. D., May, R., McKinlay, C. J. D., Thompson, B., Wouldes, T. A., & Harding, J. E. (2021). Two-year outcomes after dextrose gel prophylaxis for neonatal hypoglycaemia. *Archives of disease in childhood. Fetal and neonatal edition*, *106*(3), 278–285. https://doi.org/10.1136/archdischild-2020-320305

14. Roeper M, Salimi Dafsari R, Hoermann H, Mayatepek E, Kummer S, Meissner T. Risk Factors for Adverse Neurodevelopment in Transient or Persistent Congenital Hyperinsulinism. Front Endocrinol (Lausanne). 2020 Nov 30;11:580642. doi: 10.3389/fendo.2020.580642. PMID: 33424766; PMCID: PMC7793856.

15. Harding JE, Hegarty JE, Crowther CA, Edlin RP, Gamble GD, Alsweiler JM, et al. (2021) Evaluation of oral dextrose gel for prevention of neonatal hypoglycemia (hPOD): A multicenter, double-blind randomized controlled trial. PLoS Med18(1): e1003411. https://doi.org/10.1371/ journal.pmed.1003411

16. Kinnala, A., Rikalainen, H., Lapinleimu, H., Parkkola, R., Kormano, M., & Kero, P. (1999). Cerebral magnetic resonance imaging and ultrasonography findings after neonatal hypoglycemia. *Pediatrics*, *103*(4 Pt 1), 724–729. https://doi.org/10.1542/peds.103.4.724

17. van Kempen, A. A. M. W., Eskes, P. F., Nuytemans, D. H. G. M., van der Lee, J. H., Dijksman, L. M., van Veenendaal, N. R., van der Hulst, F. J. P. C. M., Moonen, R. M. J., Zimmermann, L. J. I., van 't Verlaat, E. P., van Dongen-van Baal, M., Semmekrot, B. A., Stas, H. G., van Beek, R. H. T., Vlietman, J. J., Dijk, P. H., Termote, J. U. M., de Jonge, R. C. J., de Mol, A. C., Huysman, M. W. A., … HypoEXIT Study Group (2020). Lower versus Traditional Treatment Threshold for Neonatal Hypoglycemia. *The New England journal of medicine*, *382*(6), 534–544. https://doi.org/10.1056/NEJMoa1905593

18. McGowan BR, Gertler TS. Hypoglycemia in Infants and Effect on Neurodevelopment. Pediatr Neurol Briefs. 2020 Dec 18;34:18. doi: 10.15844/pedneurbriefs-34-18. PMID: 33354100; PMCID: PMC7747512.

19. Masood, A., Qureshi, F., Ahmed, P., Hassan, M. U., & Ali, I. (2023). Effect of Comorbidity-free Neonatal Hypoglycemia on Neurodevelopment at 18 Months of Age: A Prospective Cohort Study. *Indian pediatrics*, *60*(11), 931–934.

20. Sivarajan, M., Schneider, J. H., Johnson, K. A., Bai, S., ElHassan, N. O., Kaiser, J. R., Nelson, D. B., Brown, L. S., Burchfield, P. J., & Brion, L. P. (2021). Decreasing early hypoglycemia frequency in at-risk newborns after implementing a new hypoglycemia screening algorithm. *Journal of perinatology : official journal of the California Perinatal Association*, *41*(12), 2840–2846. https://doi.org/10.1038/s41372-021-01263-8

21. Harris, D. L., Alsweiler, J. M., Ansell, J. M., Gamble, G. D., Thompson, B., Wouldes, T. A., Yu, T. Y., Harding, J. E., & Children with Hypoglycaemia and their Later Development (CHYLD) Study Team (2016). Outcome at 2 Years after Dextrose Gel Treatment for Neonatal Hypoglycemia: Follow-Up of a Randomized Trial. *The Journal of pediatrics*, *170*, 54–9.e92. https://doi.org/10.1016/j.jpeds.2015.10.066

## 4. Table S1: Quality assessment of Observational studies using the NOS tool for assessment of the Cohort studies

| s.no | Study ID | Selection (max 4) | | | | Outcome (max 3) | | | Comparative (max 2) | | Total Score (max 9) | Interpretation |
|---|---|---|---|---|---|---|---|---|---|---|---|---|
| | | Representativeness of the exposed cohort (1) | Ascertainment of exposure (1) | Demonstration that outcome of interest was not present at start of study (1) | Selection of non-exposed amongst cohort (i.e., sample drawn from same neonatal population/nicu) (1) | Assessment of outcome (1) | Was follow-up long enough for outcomes to occur (1) | Adequacy of follow up of cohorts (1) | Study controls for GA and Sex (1) | Study controls for additional factors (1) | | > /=6 (Good quality) 4,5 (fair quality) 1,2,3 (poor quality) |
| 1 | Brand 2004 [23] | 1 | 1 | 1 | 1 | 1 | 1 | 1 | 0 | 0 | 7 | Good |
| 2 | Duvanel 1999 [28] | 1 | 1 | 1 | 1 | 1 | 1 | 1 | 0 | 0 | 7 | Good |
| 3 | Edwards 2022 [39] | 1 | 1 | 1 | 1 | 1 | 1 | 1 | 0 | 1 | 8 | Good |
| 4 | Goode 2016 [30] | 1 | 1 | 1 | 1 | 1 | 1 | 1 | 0 | 0 | 7 | Good |

| | | | | | | | | | | | | |
|---|---|---|---|---|---|---|---|---|---|---|---|---|
| 5 | Grifffiths 1971 [24] | 1 | 1 | 1 | 1 | 1 | 1 | 0 | 0 | 0 | 6 | Good |
| 6 | Haworth 1976 [32] | 1 | 1 | 1 | 1 | 0 | 1 | 0 | 0 | 0 | 5 | Fair |
| 7 | Kaiser 2015 [29] | 1 | 1 | 1 | 1 | 1 | 1 | 1 | 0 | 1 | 7 | Good |
| 8 | Kennedy 2022 [40] | 1 | 1 | 1 | 1 | 1 | 1 | 1 | 0 | 0 | 7 | Good |
| 9 | Kerstjens 2012 [25] | 1 | 1 | 1 | 1 | 1 | 1 | 1 | 1 | 1 | 9 | Good |
| 10 | Koivisto 1972 [26] | 1 | 1 | 1 | 1 | 1 | 1 | 0 | 0 | 0 | 6 | Good |
| 11 | Mahajan 2017 [35] | 1 | 1 | 1 | 1 | 1 | 1 | 1 | 1 | 1 | 9 | Good |

| | | | | | | | | | | | | |
|---|---|---|---|---|---|---|---|---|---|---|---|---|
| 12 | Mckinlay 2015 [34] | 1 | 1 | 1 | 1 | 1 | 1 | 1 | 1 | 1 | 9 | Good |
| 13 | Mckinlay 2017 [33] | 1 | 1 | 1 | 1 | 1 | 1 | 1 | 1 | 1 | 9 | Good |
| 14 | Pildes 1974 [31] | 1 | 1 | 1 | 1 | 1 | 1 | 1 | 0 | 0 | 7 | Good |
| 15 | Qiao 2019 [37] | 1 | 1 | 1 | 1 | 1 | 1 | 1 | 0 | 0 | 7 | Good |
| 16 | Rasmussen 2020 [38] | 1 | 1 | 1 | 1 | 1 | 1 | 1 | 1 | 0 | 8 | Good |
| 17 | Stenniger 1998 [27] | 1 | 1 | 1 | 1 | 1 | 1 | 1 | 0 | 0 | 7 | Good |
| 18 | Wickstrom 2018 [36] | 1 | 1 | 1 | 1 | 1 | 1 | 1 | 1 | 0 | 8 | Good |

| | | | | | | | | | | | | |
|---|---|---|---|---|---|---|---|---|---|---|---|---|
| 19 | Shah 2022 [41] | 1 | 1 | 1 | 1 | 1 | 1 | 1 | 1 | 1 | 9 | Good |

## 5. Table S2: GRADE tables

**Summary of findings:**

### Neurodevelopmental outcomes BG <20mg/dl compared to placebo for Neurodevelopmental Outcomes

**Patient or population:** Neurodevelopmental Outcomes
**Setting:**
**Intervention:** Neurodevelopmental outcomes BG <20mg/dl
**Comparison:** placebo

| Outcomes | Anticipated absolute effects* (95% CI) | | Relative effect (95% CI) | № of participants (studies) | Certainty of the evidence (GRADE) | Comments |
|---|---|---|---|---|---|---|
| | **Risk with placebo** | **Risk with Neurodevelopmental outcomes BG <20mg/dl** | | | | |
| NDI 2-5 years | 0 per 1,000 | **0 per 1,000** (0 to 0) | **OR 0.94** (0.35 to 2.51) | 107 (2 studies) | - | |
| Epilepsy 2-5years | 0 per 1,000 | **0 per 1,000** (0 to 0) | **OR 1.24** (0.30 to 5.22) | 119 (2 studies) | - | |
| Emotional behavioural issues 2-5years | 0 per 1,000 | **0 per 1,000** (0 to 0) | **OR 0.81** (0.26 to 2.50) | 119 (2 studies) | - | |
| | | | | | | |

**Summary of findings:**

## Neurodevelopmental outcomes BG <20mg/dl compared to placebo for Neurodevelopmental Outcomes

**Patient or population:** Neurodevelopmental Outcomes
**Setting:**
**Intervention:** Neurodevelopmental outcomes BG <20mg/dl
**Comparison:** placebo

| Outcomes | Anticipated absolute effects* (95% CI) | | Relative effect (95% CI) | № of participants (studies) | Certainty of the evidence (GRADE) | Comments |
|---|---|---|---|---|---|---|
| | Risk with placebo | Risk with Neurodevelopmental outcomes BG <20mg/dl | | | | |

**Summary of findings:**

## Neurodevelopmental outcomes BG 20-34mg/dl compared to placebo for Neurodevelopmental Outcomes

**Patient or population:** Neurodevelopmental Outcomes
**Setting:**
**Intervention:** Neurodevelopmental outcomes BG 20-34mg/dl
**Comparison:** placebo

| Outcomes | Anticipated absolute effects* (95% CI) | | Relative effect (95% CI) | № of participants (studies) | Certainty of the evidence (GRADE) | Comments |
|---|---|---|---|---|---|---|
| | Risk with placebo | Risk with Neurodevelopmental outcomes BG 20-34mg/dl | | | | |

**Summary of findings:**

## Neurodevelopmental outcomes BG <20mg/dl compared to placebo for Neurodevelopmental Outcomes

**Patient or population:** Neurodevelopmental Outcomes
**Setting:**
**Intervention:** Neurodevelopmental outcomes BG <20mg/dl
**Comparison:** placebo

| Outcomes | Anticipated absolute effects* (95% CI) | | Relative effect (95% CI) | № of participants (studies) | Certainty of the evidence (GRADE) | Comments |
|---|---|---|---|---|---|---|
| | Risk with placebo | Risk with Neurodevelopmental outcomes BG <20mg/dl | | | | |
| NDI 2-5yrs | 0 per 1,000 | **0 per 1,000** (0 to 0) | **OR 2.20** (1.21 to 4.02) | 1871 (2 non-randomised studies) | ⊕⊕◯◯ Low[a] | |

| Outcomes | Exposure effect (95% CI) | № of participants (studies) | Certainty of the evidence (GRADE) |
|---|---|---|---|
| Motor Impairment | **OR 1.48** (0.98 to 2.24) | 5052 (4 non-randomised studies) | ⨁◯◯◯ Very low |
| Mild Cognitive Impairment | **OR 1.09** (0.78 to 1.52) | 1935 (4 non-randomised studies) | ⨁◯◯◯ Very low |
| Moderate-Severe Cognitive Impairment | **OR 1.26** (0.58 to 2.75) | 1935 (4 non-randomised studies) | ⨁◯◯◯ Very low |
| Epilepsy | **OR 1.93** (0.78 to 4.80) | 772 (4 non-randomised studies) | ⨁◯◯◯ Very low |
| Visual Impairment | **OR 2.15** (0.72 to 6.41) | 698 (3 non-randomised studies) | ⨁◯◯◯ Very low |
